# IDOL gene variant is associated with hyperlipidemia in Han population in Xinjiang, China

**DOI:** 10.1038/s41598-020-71241-1

**Published:** 2020-08-31

**Authors:** Dilare Adi, Jialin Abuzhalihan, Ying-hong Wang, Gulinaer Baituola, Yun Wu, Xiang Xie, Zhen-Yan Fu, Yi-Ning Yang, Xiang Ma, Xiao-Mei Li, Bang-dang Chen, Fen Liu, Yi-Tong Ma

**Affiliations:** 1 State Key Laboratory of Pathogenesis, Prevention and Treatment of High Incidence Diseases in Central Asia, Department of Cardiology, The First Affiliated Hospital of Xinjiang Medical University, Urumqi, 830054 People’s Republic of China; 2 Xinjiang Key Laboratory of Cardiovascular Disease Research, Urumqi, 830054 People’s Republic of China; 3 Health Checkup Department of the First Affiliated Hospital of Xinjiang Medical University, Urumqi, 830054 People’s Republic of China; 4 Department of General Practice, The First Affiliated Hospital of Xinjiang Medical University, Urumqi, 830011 People’s Republic of China; 5 State Key Laboratory of Pathogenesis, Prevention and Treatment of High Incidence Diseases in Central Asia, Clinical Medical Research Institute, The First Affiliated Hospital of Xinjiang Medical University, Urumqi, 830054 People’s Republic of China

**Keywords:** Dyslipidaemias, Genetic association study

## Abstract

Hyperlipidemia is one of the main risk factors that contributed to atherosclerosis and coronary artery disease (CAD). In the present study, our objective was to explore whether some genetic variants of human IDOL gene were associated with hyperlipidemia among Han population in Xinjiang, China. We designed a case–control study. A total of 1,172 subjects (588 diagnosed hyperlipidemia cases and 584 healthy controls) of Chinese Han were recruited. We genotyped three SNPs (rs9370867, rs909562, and rs2072783) of IDOL gene in all subjects by using the improved multiplex ligation detection reaction (iMLDR) method. Our study demonstrated that the distribution of the genotypes, the dominant model (AA vs GG + GA), and the overdominant model (AA + GG vs GA) of the rs9370867 SNP had significant differences between the case group and controls (all *P* < 0.001). For rs909562 and rs2072783, the distribution of the genotypes, the recessive model (AA + GA vs GG) showed significant differences between the case subjects and controls (*P* = 0.002, *P* = 0.007 and *P* = 0.045, *P* = 0.02, respectively). After multivariate adjustment for several confounders, the rs9370867 SNP is still an independent risk factor for hyperlipidemia [odds ratio (OR) = 1.380, 95% confidence interval (CI) = 1.201–1.586, *P* < 0.001]. The rs9370867 of human IDOL gene was associated with hyperlipidemia in Han population.

## Introduction

Hyperlipidemia is defined as increased levels of plasma total cholesterol (TC), low-density lipoprotein-cholesterol (LDL-C), or triglyceride (TG) and along with or without decreased level of high-density lipoprotein cholesterol (HDL-C)^1^. Hyperlipidemia is one of the main risk factors that contributed to atherosclerosis and coronary artery disease (CAD)^2,3^. Previous studies on molecular mechanisms of hyperlipidemia revealed that multiple risk factors, including genetics, high-calorie diet, age, gender, obesity, smoking, drinking, and lack of physical activity, resulted in hyperlipidemia^4–6^. Among these, genetic factors may play essential roles in formation of hyperlipidemia.

IDOL (inducible degrader of LDLR), also known as MYLIP, is an E3 ubiquitin ligase. It can stimulate the ubiquitination and degradation of the LDLR in the lysosome by interacting with its cytoplasmic domain^7^. Numerous animal studies showed that overexpression of IDOL gene in mouse liver caused the development of hypercholesterolemia and atherosclerosis^8^. Instantaneous adenoviral overexpression of mouse IDOL gene in wildtype model resulted in a significant decrease in LDLR protein and an increase in LDL-C levels^9,10^. Other possible downstream of IDOL includes VLDLR and ApoER2^11^. The upstream of IDOL is LXRs which have influence on extracellular cholesterol levels by combining with LXR element of IDOL^12^. Human genetic studies propose that IDOL may become a new therapeutic target for regulating plasma LDL-C levels. Genome-wide association study (GWAS) showed that several variants of the IDOL were correlated with plasma lipid levels^13^.

The relationship between some variants of human IDOL gene and lipid profiles was previously reported by researchers. However, their conclusions remain inconsistent. Weissglas-Volkov et al.^14^ studied IDOL gene variants and lipid levels using GWAS. Their results showed that rs9370867 single nucleotide polymorphism (SNP) of IDOL gene significantly correlated with high TC in Mexican population. However, a study in Dutch people performed by Sorrentino et al.^15^ revealed that rs9370867 variant of IDOL gene showed same frequencies between high and low LDL-C subjects. Dhyani et al.^16^ reported that the rs9370867 variant of IDOL was not associated with lipid levels in Italian population. In summary, previous reports on IDOL variants and lipid profiles still remain controversial. Thus, the purpose of the current study was to investigate the relationship between some variants of IDOL gene and hyperlipidemia in Han population in Xinjiang, China.

## Methods

### Subjects

This study was designed in a case–control study. A total of 1,172 subjects (588 diagnosed hyperlipidemia cases and 584 healthy controls) of Chinese Han were randomly selected from the First Affiliated Hospital of Xinjiang Medical University between August 2010 and October 2016. Hyperlipidemia was defined as a total plasma cholesterol > 6.22 mmol/L or low density lipoprotein cholesterol > 4.14 mmol/L or plasma triglycerides > 2.26 mmol/L and/or the current use of lipid-lowering drugs with an established diagnosis of hyperlipidemia^17^. Further, all of these subjects live in Xinjiang Uyghur Autonomous Region of China. Exclusion criteria were those suffer from impaired malignancy, connective tissue disease, concomitant valvar heart disease, renal function, valvular disease or chronic inflammatory disease, pancreatic disease, fatty liver, cirrhosis, hepatitis. Moreover, subjects also are free from thyroid disease, or any history of taking lipid-lowering drugs. The following information was collected: age, gender, blood pressure, total cholesterol (TC), triglyceride (TG), high-density lipoprotein cholesterol (HDL-C), low-density lipoprotein cholesterol (LDL-C), blood urea nitrogen (BUN), creatinine (Cr), uric acid, fasting plasma glucose (FPG).

### Genotyping

Using Haploview 4.2 software and International HapMap Project website phase I and II database (https://www.hapmap.org), we obtained three tag SNPs of IDOL: SNP1 (rs9370867), SNP2 (rs909562), and SNP3 (rs2072783) by using minor allele frequency (MAF) ≥ 0.05 and linkage disequilibrium patterns with r^2^ ≥ 0.8 as a cutoff. Blood samples were collected from all participants, and genomic DNA was extracted from peripheral blood leukocytes using a DNA extraction kit (Beijing Biotech Co. Ltd., Beijing, China). The SNP genotyping was performed using an improved multiplex ligation detection reaction (iMLDR) technique (Genesky Biotechnologies Inc., Shanghai, China). Genotyping was performed in a blinded fashion without knowledge of the patients' clinical data, and a total of 10% of the genotyped samples were duplicated to monitor genotyping quality^18^.

### Statistical analysis

Analyses were carried out using SPSS version 22.0 (SPSS, Chicago, IL). All data were assessed for normality (Kolmogorov–Smirnov test) and equal variance tests. Continuous variables are expressed as means ± SD in case of normal distribution and as the median (interquartile range) in case of non-normal distribution. Continuous variables were compared using Student's *t* test, and non-normally distributed variables were analyzed with the Mann–Whitney *U* test. Differences in enumeration data between the CAD patients and control subjects were analyzed using the Chi-squared test, as were differences in the distributions of genotypes and alleles. Logistic regression analyses were used to assess the contributions of the major risk factors. Further, linkage disequilibrium test and haplotype frequency calculation were performed using HaploView 4.2 software. Statistical power of the study was calculated through online software (https://www.stat.ubc.ca/~rollin/stats/ssize/caco.html)^19^. A *P* value < 0.05 was considered to be statistically significant^20^.

### Ethical approval of the study protocol

The study was approved by the Ethics Committee of the of the First Affiliated Hospital of Xinjiang Medical University (Xinjiang, China). All participants signed a written informed consent. The investigation was performed in accordance with the principles of the Declaration of Helsinki.

## Results

### Statistical power

Statistical power of the study was calculated through the above online software with the following parameters: an unmatched case–control study, a P_0_ value of 0.228, a RR value of 1.57, an α value of 0.05, and a sample size of 588 cases and 584 controls. The result is P = 0.93.

### Characteristics of study participants

Baseline characteristics of the included subjects were listed in Table 1. The diastolic blood pressure (DBP), uric acid levels, plasma concentration of HDL-C were similar between the case group and the control group (all *P* > 0.05). The mean age of case group was higher than the control group (*P* = 0.022). Patients with hyperlipidemia had higher levels of systolic blood pressure (SBP), BUN, Cr, FPG, TC, TG, LDL-C than control subjects (all *P* < 0.05).

**Table 1:** Clinical and metabolic characteristics of subjects.

Risk factors	Case	Control	χ^2^ or t	P value
Age (years)	58.60 ± 7.86	57.46 ± 9.26	2.289	*0.022*
Male, n (%)	216 (36.7%)	219 (37.5%)	0.074	0.809
Smoking, n (%)	260 (44.2%)	282 (48.3%)	1.952	0.178
Drinking, n (%)	199 (33.8%)	194 (33.2%)	0.051	0.853
SBP (mmHg)	127.44 ± 17.03	125.02 ± 15.19	2.568	*0.01*
DBP (mmHg)	77.03 ± 10.33	76.40 ± 9.97	1.056	0.291
BUN (mmol/L)	5.50 ± 1.62	5.29 ± 1.57	2.197	*0.028*
Cr (mmol/L)	74.85 ± 19.65	71.95 ± 17.80	2.644	*0.008*
Uric acid (umol/L)	317.37 ± 80.33	314.89 ± 81.82	0.524	0.6
FPG (mmol/L)	6.27 ± 2.83	5.67 ± 1.99	4.185	< *0.001*
TG (mmol/L)	1.82 ± 1.12	1.68 ± 0.95	2.253	*0.024*
TC (mmol/L)	4.34 ± 0.98	3.81 ± 0.87	9.800	< *0.001*
HDL-C (mmol/L)	1.10 ± 0.29	1.11 ± 0.31	0.798	0.425
LDL-C (mmol/L)	2.71 ± 0.78	2.38 ± 0.73	7.562	< *0.001*

Statistically significant values are in italics.

*SBP* systolic blood pressure, *DBP* diastolic blood pressure, *BUN* blood urea nitrogen, *Cr* creatinine, *FPG* fasting plasma glucose, *TG* triglyceride, *TC* total cholesterol, *HDL-C* high-density lipoprotein cholesterol, *LDL-C* low-density lipoprotein cholesterol.

### Distributions of genotypes and allele in cases and controls

Table 2 showed the distribution of genotypes and alleles for the three SNPs (rs9370867, rs909562 and rs2072783) of the IDOL gene. The genotype distributions of the three SNPs were in accordance with the Hardy–Weinberg equilibrium (all *P* > 0.05). For rs9370867, the distribution of the genotypes, the dominant model (AA vs GG + GA), the overdominant model (AA + GG vs GA) showed significant differences between the case subjects and the controls (all *P* < 0.001). For rs909562, the distribution of the genotypes, the recessive model (AA + GA vs GG) showed significant differences between the case subjects and the controls (*P* = 0.002 and *P* = 0.007, respectively). For rs2072783, the distribution of the genotypes, the recessive model (AA + GA vs GG) showed significant differences between hyperlipidemia patients and the control subjects (*P* = 0.045 and *P* = 0.02, respectively).

**Table 2:** Distribution of genotypes and alleles of SNPs in subjects.

Genotype	Model		Case (n, %)	Control (n, %)	OR (95%CI)	P value
**rs9370867**	Codominant	AA	377 (64.1)	451 (77.2)	1	*< 0.001*
(A > G)		GA	184 (31.3)	113 (19.3)	0.513 (0.391–0.673)	
		GG	27 (4.6)	20 (3.4)	0.619 (0.342–1.122)	
	Dominant	AA	377 (64.1)	451 (77.2)	0.527 (0.408–0.681)	*< 0.001*
		GA + GG	211 (35.9)	133 (22.8)		
	Recessive	AA + GA	561 (95.4)	564 (96.6)	0.737 (0.408–1.329)	0.372
		GG	27 (4.6)	20 (3.4)		
	Overdominant	GG + AA	404 (68.7)	471 (80.7)	0.527 (0.402–0.690)	*< 0.001*
		GA	184 (31.3)	113 (19.3)		
		A	938 (79.8)	1,015 (86.9)	1.679 (1.344–2.097)	*< 0.001*
		G	238 (20.2)	153 (13.1)		
**rs909562**	Codominant	AA	246 (41.8)	278 (47.6)	1	*0.012*
(A > G)		GA	261 (44.4)	255 (43.7)	0.865 (0.678–1.103)	
		GG	81 (13.8)	51 (8.7)	0.557 (0.377–0.823)	
	Dominant	AA	246 (41.8)	278 (47.6)	0.792 (0.629–0.997)	0.052
		GA + GG	342 (58.2)	306 (52.4)		
	Recessive	AA + GA	507 (86.2)	533 (91.3)	0.5599 (0.413–0.868)	*0.007*
		GG	81 (13.8)	51 (8.7)		
	Overdominant	GG + AA	327 (55.6)	329 (56.3)	0.971 (0.771–1.223)	0.814
		GA	261 (44.4)	255 (43.7)		
		A	753 (64)	811 (69.4)	1.267 (1.066–1.506)	*0.007*
		G	423 (36)	357 (30.6)		
**rs2072783**	Codominant	AA	245 (41.7)	268 (45.9)	1	*0.045*
(A > G)		GA	265 (45.1)	264 (45.2)	0.911 (0.714–1.161)	
		GG	78 (13.3)	52 (8.9)	0.609 (0.412–0.901)	
	Dominant	AA	245 (41.7)	268 (45.9)	0.842 (0.668–1.061)	0.158
		GA + GG	343 (58.3)	316 (54.1)		
	Recessive	AA + GA	510 (86.7)	532 (91.1)	0.639 (0.441–0.926)	*0.02*
		GG	78 (13.3)	52 (8.9)		
	Overdominant	GG + AA	323 (54.9)	320 (54.8)	1.006 (0.799–1.266)	1
		GA	265 (45.1)	264 (45.2)		
		A	755 (64.7)	800 (68.5)	1.204 (1.014–1.429)	*0.03*
		G	412 (35.3)	368 (31.5)		

Statistically significant values are in italics.

Table 3 showed the multivariable logistic regression analyses of the major confounding factors for hyperlipidemia. After multivariate adjustment for the confounders, such as age, sex, SBP, DBP, BUN, Cr, uric acid FPG, and prevalence of smoking and drinking, the rs9370867 SNP is still an independent risk factor for hyperlipidemia [odds ratio (OR) = 1.380, 95% confidence interval (CI) = 1.201–1.586, *P* < 0.001]. However, the rs909562 and rs2072783 SNPs did not represent as the independent risk factor for hyperlipidemia after multivariate adjustment [odds ratio (OR) = 1.088, 95% confidence interval (CI) = 0.956–1.238, *P* = 0.202. odds ratio (OR) = 1.032, 95% confidence interval (CI) = 0.907–1.175, *P* = 0.632, respectively].

**Table 3:** Results of logistic analysis of risk factors for hyperlipidemia.

Risk factors	OR	95% CI of OR	P value
rs9370867	1.380	1.201–1.586	*< 0.001*
rs909562	1.088	0.956–1.238	0.202
rs2072783	1.032	0.907–1.175	0.632
Age	1.003	0.988–1.018	0.685
Gender	1.004	0.722–1.397	0.979
Smoking	0.836	0.622–1.122	0.233
Drinking	1.070	0.785–1.457	0.670
SBP	1.012	1.002–1.021	*0.021*
DBP	0.975	0.961–0.990	*0.001*
BUN	1.032	0.951–1.119	0.453
Cr	1.009	1.001–1.017	*0.028*
Uric acid	0.999	0.997–1.001	0.304
FPG	1.056	0.995–1.121	0.071

Statistically significant values are in italics.

*SBP* systolic blood pressure, *DBP* diastolic blood pressure, *BUN* blood urea nitrogen, *Cr* creatinine, *FPG* fasting plasma glucose.

### The linkage disequilibrium and haplotypes for IDOL three SNPs in subjects

**Figure 1:**
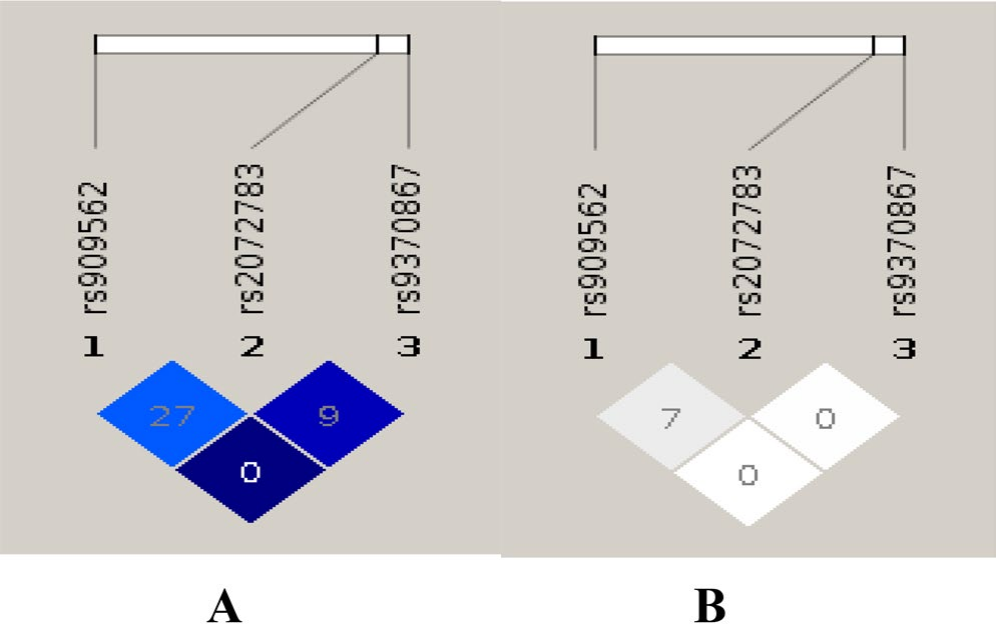
|D′| (A) and r^2^ (B) analyses of 3 SNPs in subjects.

The results of linkage disequilibrium analysis were showed in Fig. 1. Our results indicated that the two SNPs, rs909562 and rs9370867, belong to different haplotype blocks.

The haplotype frequencies of the IDOL gene in patients with hyperlipidemia and controls are shown in Table 4. The CG haplotype frequency was higher in patients with hyperlipidemia than in controls (*P* < 0.001).

**Table 4:** Haplotype distribution frequency for IDOL gene 2 SNPs.

SNPs	Haplotypes	Case group	Control group	P value	OR	95% CI
rs909562, rs2072783	AA	452 (0.7197)	508(0.7384)	–	–	–
	AG	299 (0.2955)	299 (0.2821)	0.44897	1.086338	0.8767226–1.346071
	GA	301 (0.2957)	288 (0.2722)	0.17284	1.165295	0.9352145–1.45198
	GG	120 (0.1446)	69 (0.0858)	*< 0.001*	1.623239	1.217281–2.164582

Statistically significant values are in italics.

## Discussion

In this study, we investigated the association between some variants of human IDOL gene and hyperlipidemia in Chinese Han population. This is the first attempt to study the common variants in IDOL gene and its association with hyperlipidemia in this population. Our study revealed that the rs9370867 SNP is an independent risk factor for hyperlipidemia in Han population.

IDOL gene is found to be one of the E3 ubiquitin ligases. E3 ubiquitin ligase is a kind of enzyme which can covalently combine with the substrates of various ubiquitin proteins and promotes the degradation of the substrate proteins^21^. The structure of IDOL gene can be divided into an N-terminal FERM domain and a C-terminal RING domain. The two domains are separated by a short connecting band^22,23^. In fact, IDOL showed ubiquitination activity on LDLR. Similar to other ubiquitinated proteins, the ubiquitinated LDLR is easy to degrade. Unlike other ubiquitinated proteins, which degraded in proteasome where IDOL also catalyzes its own degradation, LDLR is usually degraded in lysosomes^24,25^.

The relationship between some variants of IDOL gene and lipid profiles was first reported by Weissglas-Volkov et al.^14^ Their study revealed that the rs9370867 SNP of IDOL gene was associated with high total cholesterol in Mexican population. Further study on mechanisms showed that an A-encoding allele was associated with the more potent LDLR degradation and the low LDL uptake. In addition, a study on the same SNP performed by Sorrentino et al.^15^. In their study, they revealed that rs9370867 SNP was found to be a common variant in both high LDL-C and low LDL-C groups with similar frequencies. Another study based on a Brazilian population indicated that rs9370867 SNP was not associated with lipid levels^26^. Willer et al. summarized 157 loci associated with lipid levels in 188,578 individuals using genome-wide and custom genotyping arrays. Their study showed that the IDOL gene rs3757354 variant is correlated with serum LDL-C and TC levels^27^. Daniel et al. performed a genome-wide association study in 17,296 women from the Women’s Genome Health Study (WGHS), and indicted that the rs2480 variant of the human IDOL gene is associated with LDL-C levels^28^. Dawn et al. reported in their study conducting GWAS that the rs2142672 variant in human IDOL gene is highly associated with lipid levels^29^. Thus, this potential relationship between some variants and lipid profiles needs further investigation.

In the present study, we genotyped rs9370867, rs909562 and rs2072783 SNPs of the IDOL gene and found that rs9370867 SNP was associated with hyperlipidemia. The GA/GG genotypes of rs9370867 SNP have higher frequencies in hyperlipidemia patients than control subjects. Multivariable logistic regression analyses of the risk factors for hyperlipidemia showed that, after adjusting several confounders, the rs9370867 SNP remained as an independent risk factor for hyperlipidemia and the risk of hyperlipidemia was increased in the subjects with the G allele in rs9370867. In addition, the GA/GG genotypes of rs909562 SNP have higher frequencies in case group. However, after adjusting multiple confounders, the rs909562 did not represent as a risk factor for hyperlipidemia. Further, the GG genotype of rs2072783 has higher frequency in case subjects, but it was not an independent risk factor for hyperlipidemia after adjusting multiple confounders. The statistical power of this study is 93%. The Type I error probability associated with this test of this null hypothesis is 0.05. Therefore, the null hypothesis was rejected, and the results of this test were reliable. The linkage disequilibrium analysis showed that since the |D′| and r^2^ between rs909562 and rs9370867 are 0, the two SNPs belong to different haplotype blocks, and the correlation between rs909562 and rs2072783 was not strongly significant. However, we tried to construct haplotypes between rs909562 and rs2072783. Consequently, our results showed that there are significant differences in distributions of GG haplotype between the case and the control groups.

The limitations of the study include: first of all, the participants of our study were all from one hospital, that inevitably increases selection bias. Second, this is an observational study, and our conclusions need further investigations about specific mechanisms. Third, we have not accomplished testing and reporting the associations between the three SNPs and lipoprotein concentrations in this study. Finally, the sample size of our study was still small and based on only one center. Large sample and multi center researches are still needed to confirm our conclusions.

In summary, our study revealed that rs9370867 of the human IDOL gene was associated with hyperlipidemia in Han population. Subjects with GA/GG genotypes or G allele of rs9370867 have increased risks for hyperlipidemia.

## Data Availability

The data will not be shared, since part of the data is being reused by another study.

## Abbreviations

CAD: Coronary artery disease
HDL: High-density lipoprotein
LDL: Low-density lipoprotein
TC: Total cholesterol
TG: Triglyceride
SBP: Systolic blood pressure
DBP: Diastolic blood pressure
BUN: Blood urea nitrogen
Cr: Creatinine
FPG: Fasting plasma glucose
